# Polyphenols Modulate Alzheimer’s Amyloid Beta Aggregation in a Structure-Dependent Manner

**DOI:** 10.3390/nu11040756

**Published:** 2019-03-31

**Authors:** Huong T. T. Phan, Kaouthar Samarat, Yuzuru Takamura, Auriane F. Azo-Oussou, Yasutaka Nakazono, Mun’delanji C. Vestergaard

**Affiliations:** 1 Japan Advanced Institute of Science and Technology, 1-1 Asahidai, Nomi City, Ishikawa 923-1292, Japan; huongptt@hnue.edu.vn (H.T.T.P.); k.samarat@gmail.com (K.S.); takamura@jaist.ac.jp (Y.T.); 2 Department of Food Science and Biotechnology, Kagoshima University, 1-21-24 Korimoto, Kagoshima 890-8580, Japan; oauriane1@gmail.com (A.F.A.-O.); yasutaka33ym@gmail.com (Y.N.)

**Keywords:** polyphenols, flavonoids, trans-stilbenes, bioactivity, amyloid beta aggregation, modulation

## Abstract

Some polyphenols, which are common natural compounds in fruits, vegetables, seeds, and oils, have been considered as potent inhibitors of amyloid beta (Aβ) aggregation, one critical pathogenic event in Alzheimer’s disease (AD). However, the mechanisms by which polyphenols affect aggregation are not fully understood. In this study, we aimed to investigate the effect of two classes of polyphenols (flavonoids and stilbenes) on the self-assembly of Aβ_42, in particular, how this relates to structure. We found that the flavonoids gallocatechin gallate (GCG) and theaflavin (TF) could completely inhibit Aβ aggregation, while two stilbenes, resveratrol and its glucoside derivative piceid, could also suppress Aβ aggregation, but to a much lesser extent. Intriguingly, resveratrol accelerated the formation of Aβ fibrils before its decreasing effect on fibrillation was detected. Atomic force microscopy (AFM) images showed a huge mass of long and thin Aβ fibrils formed in the presence of resveratrol. Although the morphology was the same in the presence of piceid, the fibrils were sparse in the presence of picead. In the presence of flavonoids, Aβ morphology was unchanged from prior to incubation (0 h), in agreement with amyloid beta kinetics analysis using thioflavin-T fluorescence assay. The electrochemical data showed a higher ability of GCG and TF to interact with Aβ than resveratrol and piceid, which could be attributed to the presence of more aromatic rings and hydroxyl groups. In addition, the two flavonoids exhibited a similar propensity for Aβ aggregation, despite having some differences in their structure. However, in the case of stilbenes, the addition of a glucoside at C-7 slightly decreased anti-Aβ aggregation property compared to resveratrol. These findings contribute to a better understanding of the essential structural features of polyphenols required for inhibiting Aβ aggregation, and the possible mechanisms for modulating aggregation.

## 1. Introduction

Polyphenols are naturally occurring secondary metabolites that are found in high quantities in fruits, vegetables, seeds, oils, and other foods [1]. They play an essential role in protecting plants from ultraviolet light and against aggression by pathogens or predators, contribute to pigmentation, and facilitate growth and reproduction [2]. More than 8000 natural polyphenols have been identified. Based on the nature of their carbon skeletons, they are grouped into six main categories: flavonoids, phenolic acids and derivatives, stilbenes, curcuminoids, lignans, and tannins [3]. All polyphenols feature more than one phenolic ring with hydroxyl groups in ortho or para positions, which are necessary for redox reactions [4]. These natural compounds exhibit a strong antioxidant power due to their properties to scavenge free radicals generated by reactive oxygen species [5] and to chelate highly redox-active metal ions like iron [6,7], thus conferring a protective effect against oxidative damage [8]. Their dietary intake is significantly higher compared to other dietary antioxidants including Vitamins C and E and carotenoids [9]. Polyphenols also have anti-inflammatory effects [10]. Increasing evidence has shown that polyphenols have a beneficial impact on the prevention of diseases associated with oxidative stress such as cancer, atherosclerosis, inflammation, and neurodegenerative diseases (e.g., Alzheimer’s and Parkinson’s) [11,12,13,14].

Alzheimer’s disease (AD) is the major cause of dementia in humans, but no drug or treatment for the disease has been discovered so far. The disease is characterized by extracellular plaques of amyloid beta (Aβ) fibrils, intracellular neurofibrillary tangles of hyperphosphorylated and misfolded tau protein, vascular damage resulting from extensive plaque deposition, and the loss of neuronal cells and synapses [15]. According to the most influential hypothesis about the pathogenesis of AD, amyloid cascade, that is, the deposition of Aβ peptide into plaques in brain tissue, is the causative agent of the disease [16]. Generated from the cleavage of a single-pass transmembrane protein, amyloid precursor protein (APP), Aβ is an amphiphilic and partly folded molecule, thus being prone to self-aggregate and produce intermediate oligomers or protofibrils, and finally insoluble fibrils [17]. Aβ impairs the activity of some membrane transporters, increases cellular oxidative stress, and causes neuroinflammation, thereby inducing extensive synapse dysfunction and neuron loss [17,18]. It has been reported that the neurotoxicity of Aβ strongly depends on their aggregated state, in which oligomers and protofibrils are more toxic than soluble monomers and mature fibrils. With a high ability to interact with cell membranes, Aβ oligomers and protofibrils form pores, disrupt membrane receptors, alter membrane properties, and induce the accumulation of intraneuronal Aβ [19]. However, the exact mechanisms remain unknown. Thus, the unravelling of factors that affect Aβ aggregation is important toward the prevention and treatment of AD.

The inhibitory or modulating effects on Aβ aggregation have been found in 44 polyphenolic compounds [20]. Ono et al. (2003) reported that wine-related phenols (myrectin, morin, quercetin, kaempferol (+)-catechin and (-)-epicatechin) inhibited the formation of Aβ fibrils from fresh Aβ_40 and Aβ_42 and destabilized preformed Aβ fibrils in dose-dependent manner [21]. Similar effects of curcumin, rosmarinic acid, tannic acids, epigallocatechin gallate (EGCG), olive tree-extracted polyphenols, resveratrol, and other stilbenes were identified [22,23,24,25,26]. Furthermore, in vivo studies reported that some polyphenols such as EGCG, resveratrol, and curcumin decreased amyloid levels and plaque formation in mice brains [27,28,29]. The ability of polyphenols to prevent Aβ polymerization was proposed to be mediated by their direct interaction with Aβ or by their binding to ions, which facilitate Aβ aggregation [20]. Despite the numerous studies demonstrating the beneficial impact of some polyphenols in Aβ self-assembly, the anti-amyloidogenic properties of other various polyphenolic compounds are not fully understood. More efforts are needed in order to elucidate structure–function relationship(s).

In this study, we aimed to investigate the effect of polyphenols on the self-assembly of Aβ peptide, in particular, how this relates to structure. Two classes of polyphenols, namely, trans-stilbenes and flavonoids, were used to assess whether the gallate side chains in flavonoids or the glucoside moiety in stilbenes could provide essential insights about the interactions of structurally different polyphenols with amyloid beta peptide. For stilbenes, we chose resveratrol, the main ingredient of polyphenols in wine, and its major derivative piceid (Figure S1A,B). For flavonoids, we selected gallocatechin gallate (GCG) and theaflavin (TF) (Figure S1C,D). TF is one of the main phenolic components found in fermented black tea, whereas GCG is an epimer of EGCG, a major polyphenol in green tea and a well-known inhibitor of Aβ aggregation, but it is more stable and bioactive [30]. Resveratrol has been reported extensively in literature, thus providing us a good reference point for our current study [26,28]. As far as we are aware, there is no study about the effect of GCG on Aβ aggregation, while piceid and TF and have been reported only once [26,31]. Having structures that are closely related (between class comparison) and related at the functional group level (within class comparison) (Figure S1) enabled us to draw a structure-dependent relationship between the polyphenols and their interaction with amyloid beta. The findings of this study may help in advancing our understanding of the physicochemical mechanism involved in the bioactivity of polyphenols as potential neuro-protective agents.

## 2. Materials and Methods

### 2.1. Materials

Polyphenols of >97% purity were purchased: TF from Wako Pure Chemical (Tokyo, Japan); piceid from LKT Laboratories (Tokyo, Japan); resveratrol, GCG from Sigma-Aldrich (St. Louis, MO, USA). All other reagents were purchased from Wako Pure Chemical (Tokyo, Japan) and were of analytical grade. Deionized water obtained from Millipore Milli-Q purification system (Millipore, Bedford, MA, USA) was used for reagent preparation and for cleaning of glassware.

### 2.2. Preparation of Amyloid Beta

Aβ peptides were prepared in 0.02% ammonia solution at 0.2 mM and stored at −80 °C. Just before analysis, and after equilibration at room temperature (RT = 24 ± 1 °C), the peptides (80 µM) were diluted in 20 mM Tris-HCl buffer solution (TBS) pH 7.4, and allowed to spontaneously aggregate in TBS at 37 ± 1 °C for a specific period of time. All analyses were carried out at RT. Prior to binding assays, the Aβ solution was prepared by dilution with TBS to a final concentration of 20 µM. Each measurement was performed on a freshly extracted aliquot of the protein sample kept at 37 ± 1 °C for aggregation.

### 2.3. Preparation of Polyphenol Solutions

GCG and piceid were prepared by dissolving in Milli-Q. TF and resveratrol were prepared by dissolving in aqueous methanol. All stock solutions were made at the concentration of 1 mM and stored at −25 °C. When they were used for experiments, methanol was diluted 10 times with Milli Q water in all of them. The final working solution was 100 μM.

### 2.4. Incubation of Amyloid Beta with and without Polyphenols

Samples of Aβ (80 µM) in TBS without and with polyphenols under different concentrations were incubated in an incubator at 37 ± 1 °C. The samples were in quiescence during the incubation, except for a few seconds of mixing prior to the measurement. In order to assess the behavior of the peptide through time, we took measurements after 0 h, 12 h, and 24 h incubation. First, we investigated the behavior of Aβ incubated without polyphenols. Then, we considered various incubation times of Aβ with individual polyphenols, separately. We also checked each polyphenol’s behavior by incubating each of them separately with and without ThT. Specifically, we wanted to see if they would interact with ThT assay and cause interference. 

### 2.5. Amyloid Beta Aggregation Kinetics Using Thioflavin-T Assay 

Kinetics of Aβ fibril formation was analyzed using thioflavin T (ThT) fluorescence assay [32]. Fluorescence intensity was monitored at an excitation wavelength of 450 nm and an emission wavelength of 483 nm using a spectrofluorophotometer (FP-6500, Jasco, Tokyo, Japan). The buffer background was subtracted. The signals were collected at 0 h, 12 h, and 24 h incubation. 

The inhibition percentages on Aβ aggregation (I%) were calculated as follows [26]:

$$I\%=\frac{I_{control}-I}{I_{control}}\times 100$$

The data are expressed as means ± standard deviation (SD) for three independent experiments. Comparisons between Cu, polyphenols, and the control were performed using ANOVA analysis.

### 2.6. Morphological Observation Using Atomic Force Microscopy 

Atomic Force Microscopy (AFM) was used to image and characterize the conformation of Aβ_42 aggregates derived from the incubation of Aβ_42 alone or with lipid vesicles. In order to prepare the AFM samples, a 5 μM portion of Aβ_42 solution was uniformly spread and immobilized in a mica plate (Furuuchi Chemical Co.; Shinagawa, Tokyo, Japan). Then, the mica was washed three times with 50 μL of deionized water to exclude Tris buffer molecules and was dried under vacuum conditions. The sample was measured by AFM (SPA400-SPI 3800, Seiko Instruments Inc., Chiba, Japan) equipped with a calibrated 20 lm xy-scan, a 10 lmz-scan range PZT-scanner, and a scanning silicon nitride tip (SI-DF3, spring constant = 1.6 N/m, frequency resonance = 28 kHz, Seiko Instruments Inc.) in a dynamic force mode (DFM). All AFM operations were performed in an automated moisture control box with 30–40% humidity at room temperature. The length and height of Aβ_42 aggregates were analyzed using ImageJ and SPI software, respectively [33].

### 2.7. Characterization of Redox Activity of Polyphenols 

The electrochemical voltammetric technique was used to characterize the redox activity of polyphenols [34]. Experiments were conducted with Aβ (80 µM) incubated alone, polyphenols (80 µM) incubated alone, and Aβ incubated with polyphenols (molar ratio Aβ:polyphenols 1:1). All were incubated for 0 h, 12 h, and 24 h at 37 ± 2 °C. Differential pulse voltammetry (DPV) analyses were performed with a BDTminiSTAT100 Potentiostat (Bio-Device Technology, Inc., Ishikawa, Japan) in connection with its KME-UsbStat software. The electrode system consisted of triangular disposable electrochemical printed (DEP) chips (Bio-Device Technology, Inc.; Ishikawa, Japan) consisting of a carbon working electrode, a platinum counter electrode, and a Ag/AgCl reference electrode, connected to the mini analyzer with a measuring electrode cable. DEP chips were rinsed with PBS before each individual analysis. A volume of analyte (20 μL) was carefully dropped onto the DEP chips, and analyzed immediately. DPV parameters were as follows: scan range −0.3 V to 1.2 V; step potential 5 mV; modulation amplitude 25 mV; and scan rate 50 mV/s. 

## 3. Results and Discussion

### 3.1. Polyphenol-Modulated Amyloid Beta Aggregation 

First, we assessed how polyphenols influence Aβ_42 self-assembly using thioflavin T (ThT) assay, a common analytical method for detecting the degree of amyloid fibrillation [35]. When ThT binds to the β-sheet of Aβ_42, it shows enhanced fluorescence emission at 483 nm wavelength, instead of at 445 nm wavelength as free ThT [36]. Our previous study reported that 80 μM Aβ monomers in Tris-HCl buffer (pH 7.4) incubated at 37 °C for 12 h and 24 h spontaneously aggregated into prefibrils and fibrils, respectively [37]. Thus, we herein incubated 80 μM Aβ monomers in Tris-HCl buffer supplemented with and without polyphenols (1:1 molar ratios) for 12 h and 24 h, then analyzed the extent of amyloid fibrillation at various incubation periods based on the detected ThT fluorescent intensity at 483 nm wavelength. We also measured the aggregation extent of Aβ_42 incubated with Cu^2+^ ions, reported to inhibit Aβ fibrillation [38,39], as a positive control. 

**Figure 1:**
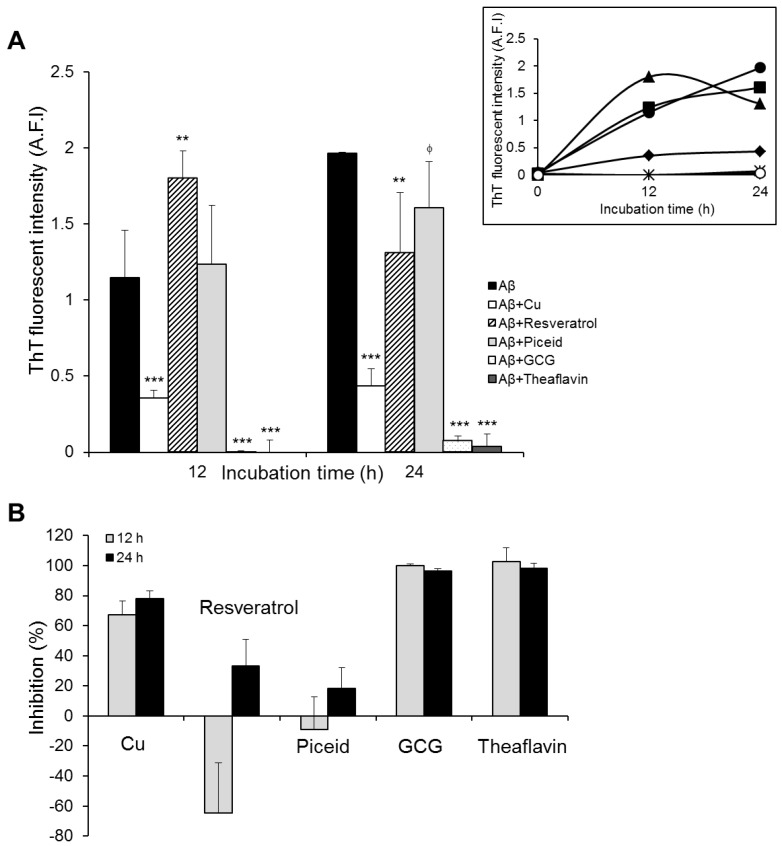
Inhibitory activity of copper ion and polyphenols on Aβ_42 aggregation. (**A**) ThT fluorescence intensity was measured after incubation of Aβ without and with Cu and polyphenols for a period of 0 h, 12 h, and 24 h (main figure). Time course curves of Aβ_42 aggregation in the absence (black, circle) and the presence of Cu (black, diamond), resveratrol (black, triangle), piceid (black, square), gallocatechin gallate (GCG) (black, star), and theaflavin (TF) (white, circle) (inserted figure). (**B**) Inhibition percentages of polyphenols on Aβ aggregation. All values represent means ± SD (*n* = 3); **, *p* < 0.01; ***, *p* < 0.001; ϕ, *p* < 0.1.

The results show that the aggregation kinetics of Aβ_42 increased steadily over the duration of the experiment (Figure 1A, insert). This was consistent with our previous studies, in which we reported that the peptide’s aggregation behavior followed a typically sigmoidal curve [33], which starts to reach the equilibrium after 24 h incubation. In agreement with previous studies, Cu^2+^ hindered Aβ_42 fibril formation, at about 70% and 80% after 12 h and 24 h incubation periods, respectively (Figure 1). The presence of polyphenols induced significant changes in the aggregation kinetics of Aβ_42. The peptide incubated with resveratrol showed a remarkable increase in ThT fluorescence compared to the control, indicating increased Aβ aggregation, at 12 h incubation. However, the fluorescence intensity decreased significantly at 24 h incubation (Figure 1A). The results indicate that resveratrol reduced the aggregation extent of Aβ in general, but it accelerated the initial aggregation of the peptide from monomeric to fibrillar species. The presence of piceid, a glucoside derivative of resveratrol, caused very little change in the aggregation of Aβ for the first 12 h. Afterwards, the aggregation kinetics was slowed down noticeably after a 24 h incubation period as shown by ThT fluorescence intensity (Figure 1A, insert). Contrary to the results with stilbenes, when Aβ was incubated with two flavonoids (GCG and TF), the fluorescence intensity of ThT was hardly observed, implying that the aggregation of Aβ was completely inhibited in the presence of the two flavonoids (Figure 1). 

These results are very interesting. They suggest a contradictory mechanism for the polyphenols’ inhibitory effects on Aβ aggregation. The two flavonoids could inhibit Aβ aggregation close to 100%. The presence of stilbenes (resveratrol and piceid) could also inhibit Aβ aggregation, but to a much lesser extent (Figure 1B). Intriguingly, resveratrol exhibited an accelerating effect on the formation of Aβ fibrils before the inhibitory activity was detected (Figure 1B). With respect to the comparison within class, hardly any difference in anti-Aβ aggregation property between the two flavonoids was observed, whereas resveratrol was slightly more anti-aggregative against the peptide than piceid but only after 24 h incubation. In order to understand more about the differences in the modulation of Aβ self-assembly by these two classes of polyphenols, we observed nanoscale morphological changes in Aβ_42 aggregates resulting from the 24 h incubation of the peptide with the polyphenols using AFM. 

### 3.2. Morphologies of Amyloid Beta Self-Assembly in the Presence of Polyphenols

**Figure 2:**
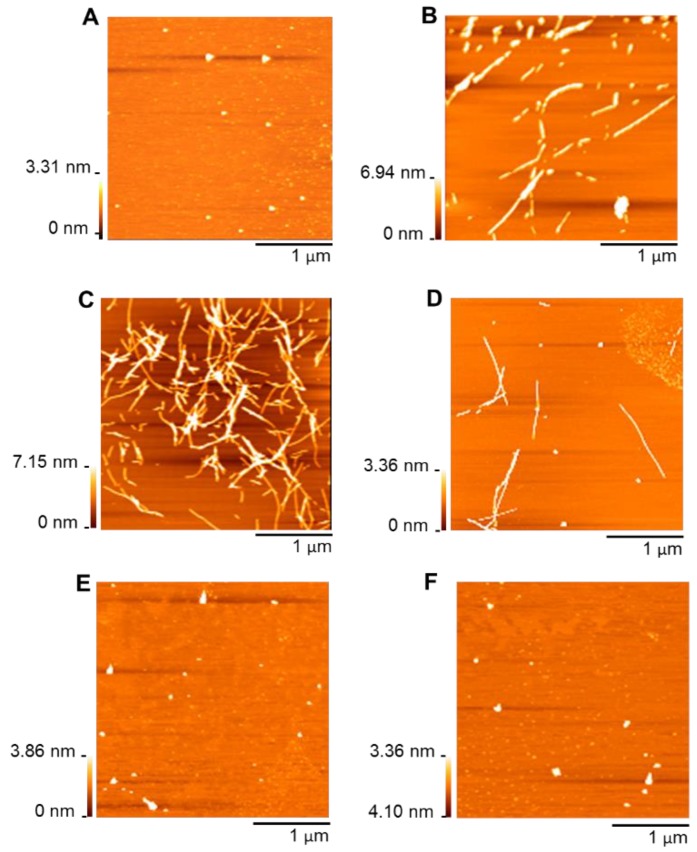
Morphology of (**A**) Aβ_42 monomers and Aβ_42 aggregates obtained after incubation (**B**) without polyphenols and with (**C**) resveratrol, (**D**) piceid, (**E**) GCG, and (**F**) TF for 24 h. The samples were analyzed using atomic force microscopy (AFM) in a dynamic force mode. All AFM operations were performed in an automated moisture control box with 30–40% humidity at room temperature. The length and height of Aβ_42 aggregates were analyzed using ImageJ and SPI software, respectively.

AFM was used to image morphologies of Aβ_42 formed in the presence and absence of polyphenols following incubation at 37 °C for 24 h. As can be seen in Figure 2, stilbenes and flavonoids induced Aβ_42 to aggregate into two different morphological species. When the peptide was incubated with stilbenes for 24 h, most of the observed aggregates had long and branched nanostructures, similar to aggregated species of the peptide by itself (Figure 2B–D). The length and height of peptide aggregates were largely in the range from 150 to 400 nm and from 2 to 6 nm, respectively (Figure S2B,C). This clearly showed that Aβ_42 was mostly fibrillar. It is interesting to note that Aβ fibrils following incubation with resveratrol were longer and more branched than those from incubation with piceid, implying a faster fibrillation of Aβ under the effect of resveratrol (Figure 2C). Conversely, the peptide did not form fibrillar aggregates in the presence of flavonoids. Only small, spherical, and unstructured particles were imaged (Figure 2E,F). Nearly 85% of these particles were 2–6 nm high and they were shorter than 100 nm (Figure S2D,E). The ability of EGCG, the epimer of GCG, and TF to promote the assembly of Aβ monomers into nontoxic, spherical, and amorphous aggregates was reported previously [31,40]. Thus, we imagine that the two flavonoids inhibited the fibril formation and induced the formation of non-toxic aggregates.

The results of the AFM experiment were consistent with our ThT assay above, indicating the strikingly different effects of two polyphenol classes, stilbenes and flavonoids, on the aggregation of Aβ_42. While the stilbenes decreased Aβ aggregation after accelerating the peptide to assemble into the less toxic fibrillary species, the latter completely hindered the fibrillogenesis. In order to investigate further the relationship between the structure of these polyphenols and their impact on Aβ aggregation, we then conducted an electrochemical analysis. We studied the interaction between the polyphenols and Aβ_42 by measuring the redox activity of polyphenols in the presence and absence of Aβ after three different incubation periods (0 h, 12 h, and 24 h). Since the antioxidant activity of polyphenols is attributed to their chemical structure, in particular the presence of aromatic OH groups, their location relative to each other, the oxidation state of the C-ring, and the overall number of OH groups present, the information could be important to understand the role of polyphenol structure in their activity on Aβ aggregation [6].

### 3.3. Redox Activity of Polyphenols in the Presence of Amyloid Beta 

**Figure 3:**
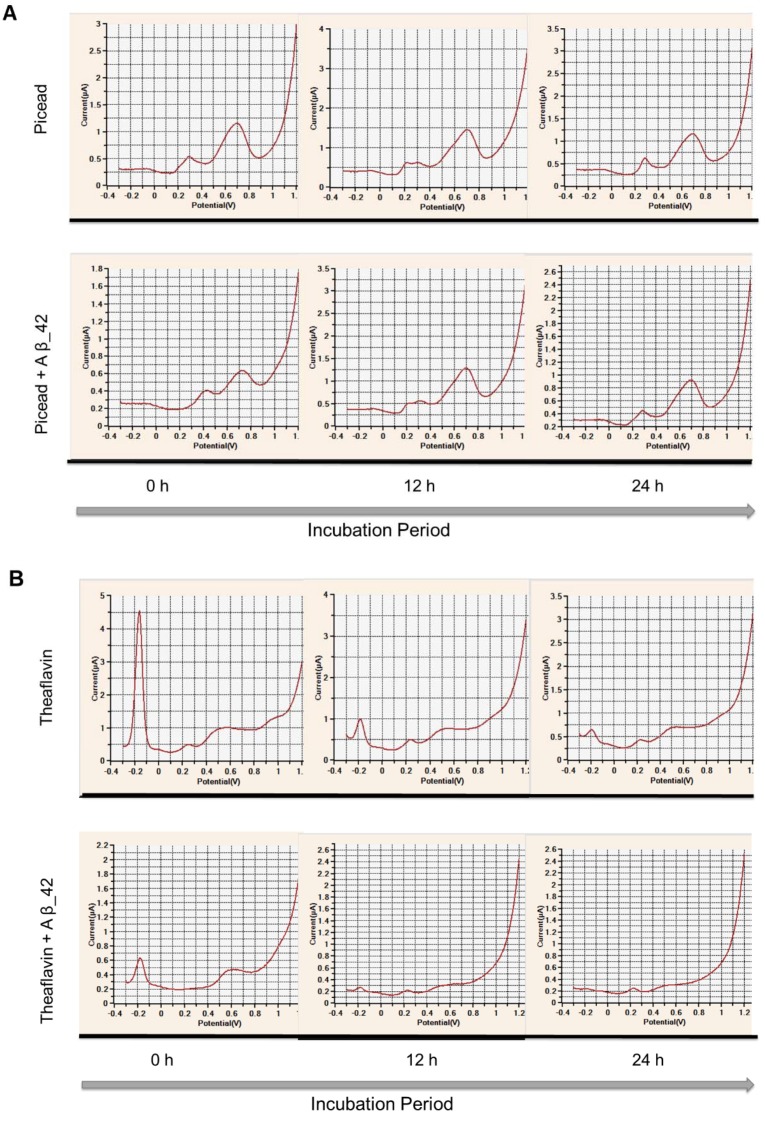
Typical voltammograms of (**A**) picead (i) and piceid plus Aβ_42 (ii); (**B**) theaflavin (TF) (i) and TF plus Aβ_42 (ii) analyzed using differential pulse voltammetry (DPV) at carbon disposal electrochemical screen-printed (DEP) chips. DPV parameters were as follows: scan range −0.3 V to 1.2 V; step potential 5 mV; modulation amplitude 25 mV; and scan rate 50 mV/s.

The electrochemical properties of GCG, TF, resveratrol, and piceid in PBS (50 mM, pH 7.4) and Aβ_42 in Tris buffer in 0 h (the control), 12 h, and 24 h incubation at 37 °C were analyzed using differential pulse voltammetry (DPV) at carbon disposal electrochemical screen-printed (DEP) chips. Typical voltammograms of TF and piceid in the presence and absence of AB are shown in Figure 3.

The number and position of peak potentials are shown in Table 1. Aβ_42 monomer displayed one peak potential at 0.71 V, which is attributed to the oxidation of tyrosine residue [41]. However, the peak potential completely disappeared after 12 h and 24 h incubation of Aβ_42 due to the assembly of the monomer to form aggregated species, in agreement with our previous study [35]. All polyphenols had at least two oxidation peak potentials, but the peak number and position varied significantly according to the class and also the particular polyphenol. 

Resveratrol and piceid showed the first peak potentials at 0.22 V and 0.29 V, respectively, corresponding to the oxidation of hydroxyl group on B-ring. The second potential of the two stilbenes appearing around 0.69 V was characterized as corresponding to the resorcinol group of resveratrol and hydroxyl groups of piceid on the A-ring. At similar conditions to our reports, we previously reported oxidation of the resorcinol group at ~0.71 V at the glassy carbon electrode [34]. In addition to the characterized peak potentials, one more oxidation peak potential was observed for resveratrol at 0.39 V. This could be explained by the further oxidation of resorcinol oxidation product(s) [42]. After 12 h and 24 h incubation at 37 °C, both stilbenes showed small changes in peak potentials. Resveratrol displayed one new peak potential at −0.25 V, whereas the peak at 0.39 V had disappeared. After 12 h incubation, piceid had a third peak potential at 0.21 V, which may correspond to the oxidation of the glucoside moiety. However, this peak potential completely disappeared after incubation for 24 h. As far as we are aware, electroanalysis of piceid has not been previously studied. 

In case of the flavonoids, GCG had two oxidation peak potentials at 0.1 and 0.59 V, corresponding to the oxidation of pyrogallol/galloyl groups in B/D-rings and the resorcinol group in the A-ring [34]. The oxidation of TF, on the other hand, was more complicated to interpret. There were six peak potentials. Based on the similarity of TF with catechin, we assume that the −0.16 V, 0.5 V, and 0.57 V oxidation potentials corresponded to its catechol groups in the fused seven-membered benzotropolone ring and C-3 OH groups of two C-rings, whereas the 0.72 V and 1.05 V peak potentials could correspond to the oxidation of resorcinol groups on the A-rings [34]. The peak potential at 0.25 V might correspond to the oxidation of the OH group in conjunction with the oxo group on the benzotropolone ring. Wu et al. suggested that the benzotropolone moiety might play an important role in the antioxidation of TF [43], and the oxidation of the C-5 OH group in conjunction with the C-4 oxo group was reported previously [34]. After 12 h and 24 h incubations, of the six oxidation potentials, only two could be observed. Meanwhile, the second oxidation peak potential for GCG could not be observed after incubation for 24 h. The changes in peak potentials of polyphenols after incubation might be due to auto-oxidation.

There were varying changes in the peak potentials of polyphenols in the presence of Aβ_42 (Table 2). Resveratrol and piceid still displayed two peak potentials, corresponding to the oxidation of the hydroxyl group on C-4′ and resorcinol groups. However, the first oxidation occurred at much higher potentials, namely, 0.45 V for resveratrol and 0.43 V for piceid, whereas the current detected for the second significantly decreased (by nearly 50% of the control, Tables S1 and S2). This indicates a decrease in the availability of functional groups to be oxidized. The incubation of stilbenes with Aβ_42 for 12 h led to the loss of the first peak potentials and the appearance of new peak potentials at 0.34 V and 0.31 V for resveratrol and piceid, respectively. This may suggest that the OH^−^ group on C-4′ had either been completely complexed with Aβ_42 and that the complex was redox-active at new potentials, or that the interaction with Aβ_42 had changed the conformation structure of the stilbenes, making the OH^−^ group on C-4′ more difficult to oxidize. Meanwhile, the second peak potential of both stilbenes shifted to slightly higher potentials. Piceid showed an additional oxidation potential at 0.22 V, which might reflect the oxidation of the glucoside group. When the incubation time increased to 24 h, the second peak potential of resveratrol could not be seen, while the oxidation potential from the glucoside moiety in piceid remained unchanged. This could imply that glucoside moiety might inhibit (perhaps due to steric hindrance) the ability of the hydroxyl group on C-5 to interact with the peptide. 

The redox activity of GCG in the presence of Aβ_42 showed a small shift in the oxidation potentials to the right, and a remarkable decrease in anodic current was detected (approximately 85% for the first and 35% for the second potential, Tables S1 and S2). After 12 h and 24 h incubation, the peak potential at 0.67 V was not observable, suggesting a possible complete complexation of resorcinol moiety and partial interaction of pyrogallol moiety/gallate side chain with Aβ_42. In the case of TF, this flavonoid showed the biggest change among the four polyphenols upon the presence of Aβ_42. It lost three oxidation potentials at 0.25, 0.5 and 0.57 V, corresponding to the oxidation of the OH group in conjunction with the oxo group on the benzotropolone ring and C-3 OH groups on two C-rings. The current detected of the first peak potentials, which reflect the oxidation of catechol, considerably dropped by approximately 85%, whereas that of the two remaining oxidation peaks corresponding to two resorcinol groups only decreased slightly. The oxidation potential of one resorcinol group disappeared and was replaced with a new potential at 0.23 V after 12 h incubation of TF with Aβ_42. When the incubation time increased to 24 h, TF lost the two last peak potentials and displayed one more new potential at 0.49 V. These results suggest that all redox-active “natural” functional groups of TF may have been directly involved in the interaction with Aβ_42 and that the interacted products of at least one of the groups were oxidized at 0.23 and 0.49 V.

### 3.4. Modulation of Amyloid Beta Aggregation by Stilbenes and Flavonoids 

The results of ThT assays and AFM experiments showed that both stilbenes and flavonoids could affect amyloid beta aggregation, but in different ways. Flavonoids (GCG and TF) could completely prevent fibril formation of Aβ_42 and remodel the peptide to assemble into non-toxic, unstructured aggregates. In comparison, stilbenes displayed a lesser anti-Aβ aggregation activity (Figure 1 and Figure 2). Previous studies have reported on the inhibitory effect of TF [31], an epi form of GCG [40], resveratrol [26,28] and piceid [26] on Aβ fibrillogenesis. However, as far as we are aware, our study is the first to report on flavonoids exhibiting a higher anti-Aβ aggregation potential than stilbenes. We investigated the structure–activity relationship of polyphenols and their effects on Aβ aggregation aside from their antioxidant properties. The main structural differences between the two classes of polyphenols are (i) the presence of an aromatic ring instead of an olefin bond for flavonoids and (ii) the presence of a gallate group on C-3 or the dimerization at B-rings producing a fused seven-membered benzotropolone ring and additional A- and C-rings for the flavonoids. This means that flavonoids possess more aromatic rings and hydroxyl groups than stilbenes, which most likely confers on them their higher anti-aggregative activity against Aβ compared to the latter. Numerous studies have showed that anti-aggregation compounds can exert their effects by forming covalent and non-covalent interactions such as π–π stacking interactions, hydrogen bonding, or charge–charge interactions between an inhibitor and the backbone of side-chain residues of the target protein. The presence of rings in the chemical structure of polyphenols enables them to form covalent interactions with hydrophobic amino-acid residues (Tyr, Phe), whereas hydroxyl groups bind to hydrophilic amino-acid residues (His6, Ser8, Tyr10, His14, Lys16) of Aβ [20,44,45]. Our electrochemistry data indicated a higher ability of flavonoids to interact with the peptide compared to stilbenes. Pyrogallol moiety/gallate side chain of GCG and catechol of TF are the most potent group binding to Aβ, as demonstrated by a dramatic plunge in the current detected for their oxidation potentials (about 85% upon 0 h incubation, Tables S1 and S2). TF lost three oxidation potentials at 0 h incubation with Aβ, and the potentials of the other oxidizable groups could not be observed when the incubation time increased, suggesting that all functional groups of the flavonoid interacted completely with the peptide (Table 2). In addition to a high number of aromatic rings and hydroxyl groups, the presence of phenolic rings in all sides of GCG and TF molecules (Figure S1C,D) is thought to form a “hydroxyl edge” on each side of polyphonic compounds that might improve binding to polypeptide chains [44]. On the other hand, the opening of the benzyl ring forms a linear shape in stilbenes with two hydrophilic ends and the central hydrophobic region (Figure S1A,B), likely reducing the stilbenes’ interactive ability with Aβ, relative to flavonoids. 

Of additional interest is the faster Aβ aggregation in the presence of resveratrol, during the first 12 h incubation, followed by a significant decrease of fibrillation extent at 24 h incubation compared to the control (Figure 1). This suggests the ability of resveratrol to induce a remarkably faster formation of Aβ fibrils during the first 12 h incubation, before its decreasing effect on Aβ aggregation was detected. Although much has been studied using resveratrol, the anti-aggregative property of this polyphenol against Aβ remains complicated to interpret. Most of the studies reported that resveratrol is able to inhibit Aβ aggregation. However, Feng et al. (2009) demonstrated that the phenol could not prevent oligomer formation although this group detected its positive effect on the polymerization of Aβ_42 monomers and the destabilization of Aβ_42 fibrils [45]. According to Ladiwala et al. (2010), resveratrol selectively remodeled Aβ soluble oligomers, fibrillary intermediates, and fibrils into non-toxic, high molecular weight, and unstructured aggregates, but it did not alter the aggregation behavior of freshly dissolved Aβ monomer. The authors suggested that resveratrol may promote conversion of soluble oligomers into either fibrillary intermediates or fibrils, and then remodel a common Aβ conformation into large, unstructured aggregates [46]. Based on previous studies and our results, we propose that resveratrol affects Aβ aggregation through two main steps: (i) accelerate a fast conversion of Aβ monomers into fibrillary species, which are less toxic and elicit more the phenol to remodel their structure [46]; (ii) remodel the structure of Aβ fibrils into non-toxic, unstructured aggregates, resulting in the reduction of fibrillation extent. The mechanism by which the stilbene induces a fast formation of Aβ fibrils before suppressing fibrillation is still unclear. One possibility involves the molecular conformation of resveratrol. It has been reported that the driving force bringing Aβ nuclei together during aggregation process are non-native side-chain electrostatic and hydrophobic interactions [47]. Since resveratrol exhibits a central hydrophobic region and a small, linear shape (Figure S1A), it would be able to mediate the nucleation from Aβ monomers via hydrophobic interaction, thereby accelerating the formation of fibrils. When Aβ fibrils are formed, aromatic staking, which is produced by the self-stacking of aromatic residues in amyloid core, might elicit resveratrol remodeling activity through interaction with the phenolic ring of the polyphenol, thus reducing the degree of aggregation [46].

Another important finding of our work is how changes in functional groups, within each class, have an impact on their effect on Aβ aggregation. Specifically, we have semi-characterized the anti-amyloidogenic activities of polyphenols, at the functional group level. The presence of the gallate moiety on C-3 or the dimerization at B-rings producing a fused seven-membered benzotropolone ring and additional A- and C-rings (Figure S1C,D) appeared not to have influenced the anti-aggregative potency of flavonoids against Aβ. Although electrochemistry data indicated a higher ability of TF to interact with Aβ_42 than GCG (Table 2), there was no difference in their effect on Aβ aggregation, observed by ThT and AFM experiments (Figure 1 and Figure 2E,F). This could imply that the electrochemical data may be more sensitive and provide more detailed information on the interaction between the peptide and the flavonoids. However, it is premature to draw that conclusion and further studies are required to confirm or refute it. The similarity between GCG’s and TF’s effects (AFM and ThT assay results) are in good agreement with Grelle et al.; who reported that TF and EGCG, an epi form of GCG, exhibit a similar propensity for Aβ fibrillogenesis [31]. Another possibility would be that the differently anti-aggregative activities of the two flavonoids occurred in a shorter incubation time (less than 12 h), so that they could not be detected in this study. Conversely, the addition of a sugar group on C-7 (Figure S1A,B) decreased the ability of piceid to inhibit amyloid beta aggregation, compared to resveratrol, which has a hydroxyl group instead, perhaps due to steric hindrance. The extent Aβ fibrillation detected from 24 h incubation with piceid is slightly smaller than that from 24 h incubation with resveratrol, and the formation of Aβ fibrils during 12 h incubation in the presence of piceid was relatively slow (Figure 1 and Figure 2C,D). Similarly, using UV–Visible spectrophotometry, Rivière et al. (2007) showed resveratrol to have a slightly higher inhibitory activity on Aβ aggregation in comparison with piceid [26]. Our electrochemistry data suggested that the change in the anti-Aβ aggregation property of piceid might be attributed to the existence of glucoside on C-7 of the A-ring. Piceid displayed the peak potentials corresponding to the oxidation of glucoside and OH^−^ groups on the A-ring during all incubation periods with Aβ, whereas the oxidation potential of resorcinol group on the A-ring of resveratrol disappeared after 24 h incubation (Table 2). This suggests that the resorcinol group in resveratrol could interact completely with the peptide, whereas the interacting ability of the functional group on the A-ring of piceid was attenuated by the presence of glucoside. 

## 4. Conclusions

In this paper, we conducted three experiments to investigate the modulating effect of polyphenols (flavonoids and stilbenes) on Alzheimer’s Aβ_42 aggregation. The results have shown the two polyphenol classes to inhibit Aβ aggregation in different ways. On the one hand, flavonoids completely inhibited fibrillation of Aβ monomers and induced the formation of spherical, unstructured aggregates. ThT assay results showed that both flavonoids exhibited a similar propensity for anti-Aβ aggregation, regardless of some differently functional groups, in agreement with the AFM analysis. Electroanalysis of the interaction provided more light on the matter and suggested TF to have a higher interaction with the peptide than GCG. On the other hand, stilbenes were able to suppress Aβ aggregation but to a significantly lesser extent. Interestingly, resveratrol, a common stilbene, accelerated the formation of Aβ fibrils during the first 12 h incubation before decreasing the aggregation of the peptide. AFM images showed a huge mass of long and thin Aβ fibrils formed in the presence of resveratrol. Although the morphology was the same in the presence of piceid, the fibrils were sparse. Electrochemical analyses indicate that resveratrol does have a higher degree of interaction with Aβ than does piceid. It is noteworthy that our results clearly demonstrate the importance of multi-analyses in order to understand better what could otherwise be interpreted as subtle differences, if not incorrectly. For example, the AFM images of Aβ fibrils at 24 h in the presence of resveratrol, on their own, could be misinforming. 

We attributed the differences in bio-activities to the main structural differences between the two classes. The presence of an aromatic ring instead of an olefin bond and the substitution of the gallate group for C-3 hydroxyl or the dimerization at B-rings producing a fused seven-membered benzotropolone ring and additional A- and C-rings for the flavonoids result in a higher number of aromatic rings and hydroxyl groups in flavonoids than in stilbenes, thereby increasing the ability of the former class to interact with Aβ. In the comparison within classes, two flavonoids, GCG and theaflavin, appear to have a similar propensity for anti-Aβ aggregation, although the electrochemical data show TF to be more interactive with Aβ that GCG. However, the presence of glucoside on C-7 slightly reduced the inhibitory property of piceid, compared to resveratrol. These results are important to help unravel the potential of these polyphenols as Aβ aggregation inhibitors, at a functional group level, which further opens the key to the tailored design of bioactive compounds as potential drug candidates.

## Figures and Tables

**Table 1:** Peak potentials of polyphenol (80 μM) solutions incubated at 37 °C for 0 h, 12 h, and 24 h.

Compounds	Peak Potentials (V)													
	0 h							12 h				24 h		
	1	2	3	4	5	6	7	1	2	3	4	1	2	3
GCG	0.1					0.59		0.11			0.6	0.13		
TF	−0.16	0.25		0.5	0.57	0.72	1.05	−0.18		0.24	0.56	−0.19	0.23	0.54
Resveratrol		0.22	0.39			0.69		−0.25		0.35	0.7	−0.21	0.31	0.67
Piceid		0.29				0.7			0.21	0.31	0.7		0.28	0.69
Aβ_42						0.71								

**Table 2:** Peak potentials of polyphenol (80 μM) solutions incubated with Aβ_42 at 37 °C for 0 h, 12 h, and 24 h.

Polyphenols	Potentials (V)											
	0 h				12 h				24 h			
	1	2	3	4	1	2	3	4	1	2	3	4
GCG	0.14		0.67		0.11				0.11			
TF	−0.18		0.63	1.07	−0.18		0.23	0.66		0.23	0.49	
Resveratrol		0.45	0.66				0.34	0.71			0.35	
Piceid		0.43	0.73			0.22	0.31	0.7			0.27	0.69

## Supplementary Materials

The following are available online at https://www.mdpi.com/2072-6643/11/4/756/s1, Figure S1: Chemical structure of (A) resveratrol, (B) piceid, (C) gallocatechin gallate, and (D) theaflavin, Figure S2: (i) Length and (ii) height distribution of Aβ_42 aggregates obtained from 24 h incubation (A) without polyphenols and (B) with resveratrol, (C) piceid, (D) gallocatechin gallate, and (E) theaflavin. Aβ_42 was incubated at 80 μM in Tris buffer (20 mM, pH 7.4). The samples were using AFM in a dynamic force mode. All AFM operations were performed in an automated moisture control box with 30–40% humidity at room temperature. The length and height of Aβ_42 aggregates were analyzed using ImageJ and SPI software, respectively, Table S1: The current detected for peak potentials of polyphenol (80 μM) solutions incubated at 37 °C for 0 h, 12 h, and 24 h, Table S2: The current detected for peak potentials of polyphenol (80 μM) solutions incubated with Aβ_42 at 37 °C for 0 h, 12 h, and 24 h.
