# Supplementary material for: Polyphenols Modulate Alzheimer’s Amyloid Beta Aggregation in a Structure-Dependent Manner
Source: Nutrients. 2019 Mar 31;11(4):756. doi: 10.3390/nu11040756 (PMC6521010; doi:10.3390/nu11040756)
Supplement: Supplementary file 1 [file nutrients-11-00756-s001.pdf]

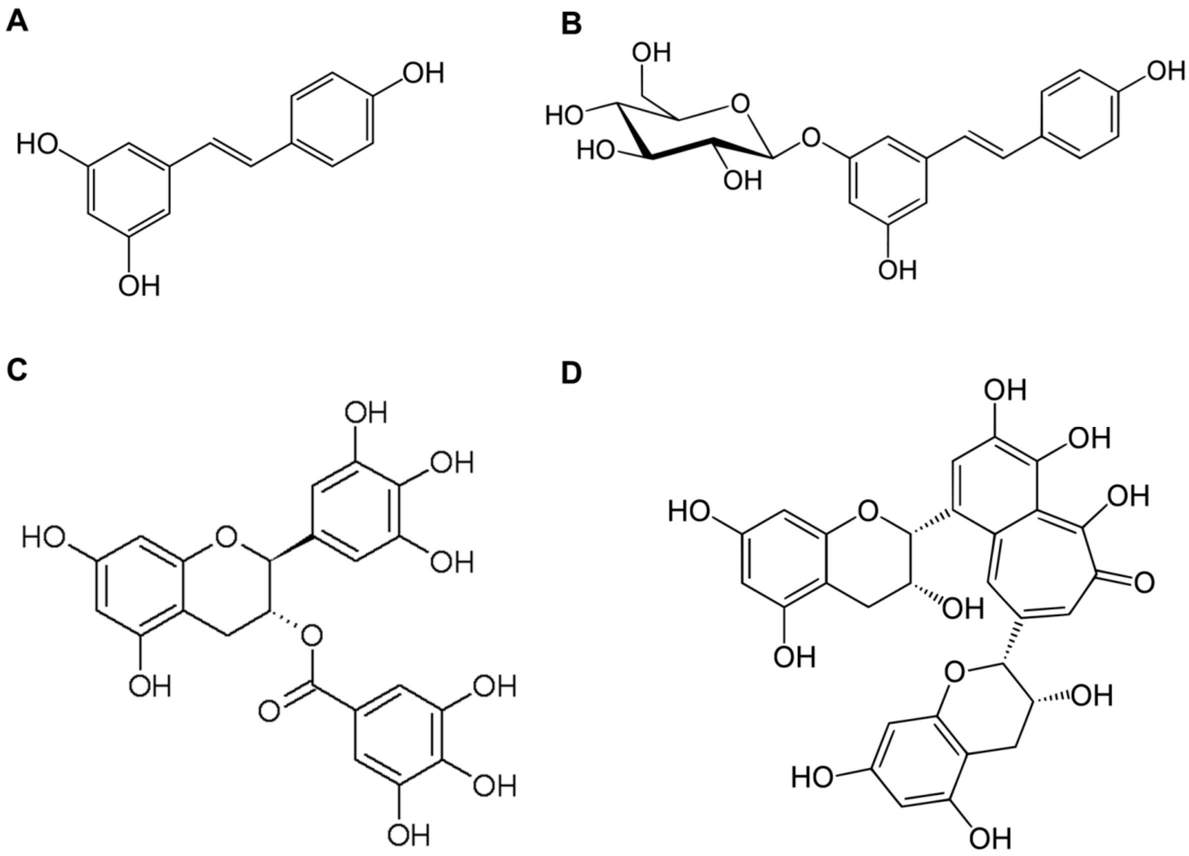

Figure S1. Chemical structure of resveratrol (A), piceid (B), gallocatechin gallate (C) and theaflavin (D).

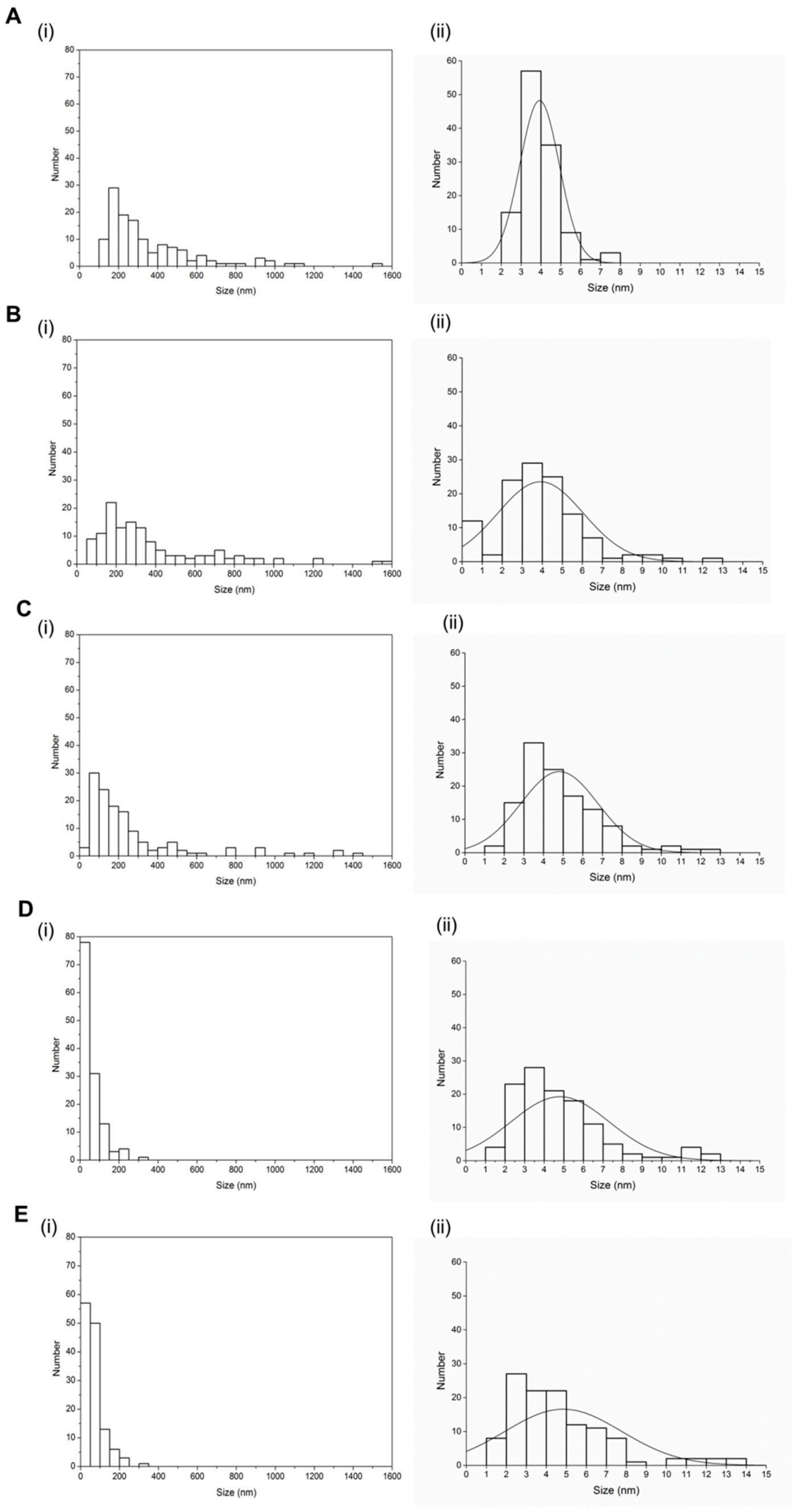

Figure S2. (i) Length and (ii) height distribution of Aβ_42 aggregates obtained from 24 h incubation (**A**) without polyphenols and with (**B**) resveratrol, (**C**) piceid, (**D**) gallocatechin gallate and (**E**) theaflavin. Aβ_42 was incubated at 80 μM in Tris buffer (20 mM, pH 7.4). The samples ware using atomic force microscopy (AFM) in a dynamic force mode. All AFM operations were performed in an

automated moisture control box with 30–40% humidity at room temperature. The length and height of Aβ_42 aggregates were analysed using Image J and SPI software, respectively.

Table S1. The current detected for peak potentials of polyphenol (80 μM) solutions incubated at 37 °C for 0 h, 12 h and 24 h.

**Compounds**	**Current (μA)**													
	**0 h**							**12 h**				**24 h**		
	**1**	**2**	**3**	**4**	**5**	**6**	**7**	**1**	**2**	**3**	**4**	**1**	**2**	**3**
GCG						0.7		0.65			0.44	0.5		
Theaflavin	1.86	0.54		1	1.04	0.97	1.43	0.97		0.49	0.77	0.64	0.45	0.72
Resveratrol	1.43	0.75	1.05			1.43		1.13		0.59	0.83	0.45	0.38	0.62
Picied		0.53				1.15			0.65	0.65	1.46		0.61	1.18
Aβ-42						0.29								

Table S2. The current detected for peak potentials of polyphenol (80 μM) solutions incubated with Aβ_42 at 37 °C for 0 h, 12 h and 24 h.

**Polyphenols**	**Current (μA)**											
	**0 h**				**12 h**				**24 h**			
	**1**	**2**	**3**	**4**	**1**	**2**	**3**	**4**	**1**	**2**	**3**	**4**
GCG	0.28		0.45		0.38				0.33			
Theaflavin	0.64		0.49	0.99	0.28		0.24	0.34		0.26	0.3	
Resveratrol		0.61	0.77				0.36	0.59			0.36	
Picied		0.42	0.64			0.53	0.58	1.3		0.47		0.93
